# Allergen particle binding by human primary bronchial epithelial cells is modulated by surfactant protein D

**DOI:** 10.1186/1465-9921-11-83

**Published:** 2010-06-22

**Authors:** Carsten Schleh, Veit J Erpenbeck, Carla Winkler, Hans D Lauenstein, Matthias Nassimi, Armin Braun, Norbert Krug, Jens M Hohlfeld

**Affiliations:** 1 Fraunhofer Institute for Toxicology and Experimental Medicine, Nikolai-Fuchs-Str. 1, Hannover, Germany; 2 Hannover Medical School, Hannover, Germany; 3 Technical University Carolo-Wilhelmina Braunschweig, Braunschweig, Germany; 4 Comprehensive Pneumology Center, Institute of Lung Biology and Disease, Helmholtz Zentrum München - German Research Center for Environmental Health, Neuherberg, Germany

## Abstract

**Background:**

Allergen-containing subpollen particles (SPP) are released from whole plant pollen upon contact with water or even high humidity. Because of their size SPP can preferentially reach the lower airways where they come into contact with surfactant protein (SP)-D. Our previous work demonstrated that SP-D increases the uptake of SPP by alveolar macrophages. In the present study, we investigated the uptake of SPP in human primary epithelial cells and the potential modulation by SP-D. The patho-physiological consequence was evaluated by measurement of pro-inflammatory mediators.

**Methods:**

SPP were isolated from timothy grass and subsequently fluorescently labelled. Human primary bronchial epithelial cells were incubated with SPP or polystyrene particles (PP) in the presence and absence of surfactant protein D. In addition, different sizes and surface charges of the PP were studied. Particle uptake was evaluated by flow cytometry and confocal microscopy. Soluble mediators were measured by enzyme linked immunosorbent assay or bead array.

**Results:**

SPP were taken up by primary epithelial cells in a dose dependent manner. This uptake was coincided with secretion of Interleukin (IL)-8. SP-D increased the fraction of bronchial epithelial cells that bound SPP but not the fraction of cells that internalized SPP. SPP-induced secretion of IL-8 was further increased by SP-D. PP were bound and internalized by epithelial cells but this was not modulated by SP-D.

**Conclusions:**

Epithelial cells bind and internalize SPP and PP which leads to increased IL-8 secretion. SP-D promotes attachment of SPP to epithelial cells and may thus be involved in the inflammatory response to inhaled allergen.

## Background

Allergen-containing subpollen particles (SPP) are released from whole plant pollen upon contact with water or in the presence of high humidity [1]. This release occurs regularly in our environment and it is associated with an increased occurrence of asthma-symptoms during thunderstorms [1]. Because of their size (d < 5 μm) SPP may reach the lower airways of the lung where they hit the epithelial lining fluid [2]. Since the epithelial lining fluid is covered with the pulmonary surfactant layer, surfactant is the first structure which comes into contact with inhaled SPP or other particles [3,4]. The surfactant allows the impingement of the particles which means that they are displaced from the airspace to the aqueous hypophase due to wetting forces [5,6]. In the aqueous hypophase, SPP may get into contact with epithelial cells and also interact with surfactant components like surfactant protein (SP) -D [7]. SP-D belongs to the family of collectins (collagen containing lectins) and is build of 12 monomers of 43 kDa which each consists of an N-terminal region, a collagen-like domain, a neck region and a globular head carbohydrate recognition domain (CRD) [7]. Via the CRD SP-D can bind to SPP as well as to various pathogens which may lead to an increased phagocytosis by alveolar macrophages [8,9].

In inflammatory lung diseases, airway epithelial cells act as important immunomodulators [10] and by interaction with inhaled allergen, they may play an essential role in allergic asthma [11]. Thereby, epithelial cells can secrete various cytokines like interleukins -5, -8 or -13 [12-14]. So far, only few data exist about the interaction of inhalable allergen particles with airway epithelial cells and surfactant components. In this study, the interaction of SPP, isolated from timothy grass, with human primary bronchial epithelial cells and its modulation by surfactant protein D was investigated. In addition, the alveolar epithelial cell line A549 was used to investigate the SP-D effect on allergen particle binding and uptake in comparison to primary bronchial cells because A549 cells are often used as a model for airway epithelial barrier cells. Finally, in order to study the influence of size and surface charge on particle binding and uptake, experiments with various polystyrene particles of different size and surface charge were performed.

## Methods

### Material

Rat recombinant SP-D (SP-D) was purified by maltose affinity chromatography from the media supernatant of cultured Chinese hamster ovary cells stably transfected with a full-length rat SP-D cDNA clone as described previously [15]. Timothy grass (*Phleum pratense*) pollen were obtained from Allergon (Ängelholm, Sweden). Polystyrene particles in three different sizes (0.5 μm - 1 μm - 3 μm) were purchased from Polysciences (Eppelheim, Germany). In addition, polystyrene particles with different surface charges (positive - negative - plain) were obtained from Invitrogen (Karlsruhe, Germany). All other reagents, unless otherwise specified, were purchased from Sigma Chemical (Deisenhofen, Germany).

### Subpollen Particles

SPP were isolated from timothy grass pollen as described previously [8]. Briefly, 300 mg of pollen were shaken and vortexed in 40 ml of deionized, autoclaved water for 3 min. Whole pollen and pollen fragments were separated by centrifugation at 50 × g for 4 min. The supernatant was filtered (5 μm filter, VWR International, Hannover, Germany) and centrifuged twice at 2500 × g for 10 min. The resulting pellet was either resuspended in 1 ml of sterile NaHCO_3 _(0.1 M) for fluorescence labelling or resuspended in phosphate buffered saline (PBS) for direct use in the experiment. To determine the number of SPP, an aliquot was diluted in PBS (1:100) and counted in an improved Neubauer chamber.

Immediately after the isolation procedure, SPP were fluorescently labelled with Alexa Fluor 488 fluorescent dye (Molecular Probes, Eugene, OR). For the staining procedure an amount of 1 × 10^9 ^- 2 × 10^9 ^SPP in 1 ml NaHCO_3 _(0.1 M) was used. The suspension was transferred into the vial of reactive dye and rotated for 1 h at room temperature in the dark. Afterwards 14 ml sterile PBS were added, centrifuged at 2500 × g for 12 min and the pellet was resuspended in 1 ml of PBS. The SPP were counted under fluorescence light in an improved Neubauer chamber.

### Particle characterisation

Diameters of SPP were analyzed by a Cambridge Stereoscan 360 scanning electron microscope (SEM). The diameters of approximately 300 SPP were measured.

The zeta potential was measured by laser doppler anemometry (LDA) using a Zetasizer ZS Nano (Malvern Instruments, Herrenberg, Germany). The analysis was performed at a temperature of 20°C using samples at appropriate working dilution. All measurements were carried out in triplicates.

### Normal human bronchial epithelial cells

Normal human bronchial epithelial cells (NHBE), derived from non-smoking subjects, were obtained from Lonza (Basel, Switzerland). Cells were delivered on dry ice and subsequently stored in the gas phase of liquid nitrogen or used immediately after receipt. NHBE were cultured in bronchial epithelial basal medium added with supplement mix (Lonza, Basel, Switzerland) at 37°C and 5% CO_2 _in cell culture flasks (25 cm^2 ^growth area) and grown to 80% confluency. Cells from passage 1 were seeded (3500 cells/cm^2^) in wells of a 24-well plate and cultured to confluency. Finally, medium was replaced, fluorescently labelled SPP with or without surfactant proteins were added and subsequently incubated at 37°C and 5% CO_2 _for 8 hours. In additional experiments, NHBE were incubated for 8 hours with unlabelled SPP with or without SP-D and the supernatant was stored at -80°C for determination of soluble mediators.

### Human primay bronchial epithelial cells - Fiberoptic Bronchoscopy

Human primary bronchial epithelial cells (HPBEC) were obtained from healthy non-smoking subjects by bronchial brushing using a fiberoptic bronchoscope (BF160, Olympus Optical, Hamburg, Germany). The bronchoscopic procedere has been described before [16]. Epithelial cells were obtained using a standard sterile single-sheathed nylon cytology brush (Olympus Cytology Brush 5 mm, Hamburg, Germany). This was passed through the bronchoscope channel into the lower airways. Two brushings were sampled from the bronchial mucosa of the forth- and fifth-generation bronchi. Cells were removed from the brush with 30 ml 0.9% sterile NaCl, placed in a 50 ml tube and shortly vortexed. Cells were sedimented at 250 g for 10 minutes at 4°C. The pellet was resuspended in 2 ml bronchial epithelial basal medium plus supplement mix (Lonza, Basel, Switzerland). Two ml Dispase II (Roche Diagnostics, Mannheim, Germany) were added. Finally, cells were counted in an improved Neubauer chamber and seeded in 6-well-plates in 3 ml medium. After reaching 80% confluency, cells were harvested by Trypsin/EDTA and seeded in 24 well plates (3500 cells/cm^2^) and cultured to confluency. Afterwards, the medium was replaced and the particles as well as surfactant proteins were added. The plates were incubated for 8 hours at 37°C and 5% CO_2 _and cells were subsequently evaluated by flow cytometry.

The Hannover Medical School ethics committee approved the study protocol. Each subject signed a written informed consent before being included in the study.

### A549 cells

A549 cells were obtained from the American Type Culture Collection (ATCC) and cultured in RPMI 1640 Medium (Cambrex Bio Sciences, Walkerswille, MD) supplemented with 10% heat-inactivated FCS and 1% penicillin-streptomycin in a 37°C humidified atmosphere with 5% CO_2_. 1 × 10^6 ^cells of passage 10 - 60 were seeded in 1 ml medium in a 24 well plate and incubated at 37°C and 5% CO_2 _and cultured to confluency. Afterwards, the medium was replaced and the SPP or PP as well as surfactant proteins were added. The plates were incubated for 8 hours at 37°C and 5% CO_2 _and cells were subsequently evaluated by flow cytometry.

### Cell labelling and fixation

Cultured cells were washed with HEPES buffered saline solution (Lonza, Basel, Switzerland), harvested with trypsin/EDTA solution (Lonza, Basel, Switzerland) and placed in polypropylene tubes (Becton Dickinson, Heidelberg, Germany). Cells were centrifuged at 300 g for 10 min. After removal of the supernatant, cells were fixed in paraformaldehyde (4%) for 30 min in the dark. After a second centrifugation (300 g, 10 min), the supernatant was discarded and cells were resuspended in PBS with TO-PRO-3 iodide (1:500; Molecular Probes, Eugene, OR) for nuclei staining. After 30 min incubation at 4°C, cells were centrifuged at 300 g for 10 min and cell pellets were resuspended in 400 μl PBS for flow cytometric analysis.

### Flow Cytometry

Cells were analyzed by flow cytometry with a Coulter FC-500 (Beckmann Coulter, Krefeld, Germany) equipped with a 488- nm argon-ion laser and a 635- nm red diode laser. A minimum of 5000 cells (gated on positive nuclear staining) was counted from respective samples. The percentage of cells that participated in uptake or attachment of SPP was calculated as the percentage of positive cells in relation to the total number of cells analyzed. Mean fluorescence intensity (MFI) of positive cells was evaluated to determine the relative amount of SPP that were bound or internalized.

### Confocal Microscopy

Confocal microscopy was performed with a Zeiss LSM 510 META run by LSM 510 software (Zeiss, Oberkochen, Germany). To determine whether the fluorescently labelled SPP were attached to the surface or located inside the cells, three-dimensional images were taken. Cell cytoskeleton was stained with an F-Actin antibody (Rhodamine phalloidin, Molecular Probes, Eugene, USA, 1:100) and a nucleus staining was obtained with TO-PRO-3 iodide. At least ten three-dimensional-pictures of each cell culture in chamber slides with SPP alone or in the presence of SP-D were taken and about 30 cells per picture were counted for the evaluation.

### Analysis of soluble mediators

Supernatants of NHBE, treated with unlabelled SPP or PP, were analyzed for CXCL8/Interleukin 8 by enzyme linked immunosorbent assay (IL-8 Duoset; R&D Bioscience, Wiesbaden, Germany) and for the mediators Eotaxin, MCP-1, IL-1α, RANTES and GM-CSF with a Bioplex Protein Array System (Biorad, Munich, Germany) and a Milliplex bead kit (Millipore, Schwalbach, Germany) according to the manufacturer's instructions.

### Statistical Analysis

Values are given as means ± SEM. Statistical analysis was performed using GraphPad Prism^®^, Version 4.03. Statistical comparison of the means was performed by ANOVA, followed by a post hoc Dunnet or Tuckey test. For comparison of two groups, the t-test was used. P-values < 0.05 were considered to be significant.

## Results

### Characterization of Subpollen particles

**Figure 1:**
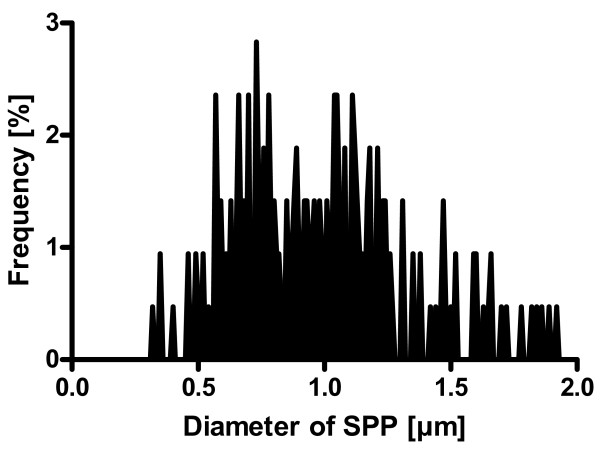
**Diameter distribution of SPP**. Approximately 300 SPP were analysed by scanning electron microscopy. The frequency distribution of diameters is shown.

Subpollen particles, isolated from pollen of *Phleum pratense *were between 0.3 μm and 1.9 μm in diameter (Figure 1). The mean diameter was 0.99 μm.

### Particle uptake

**Figure 2:**
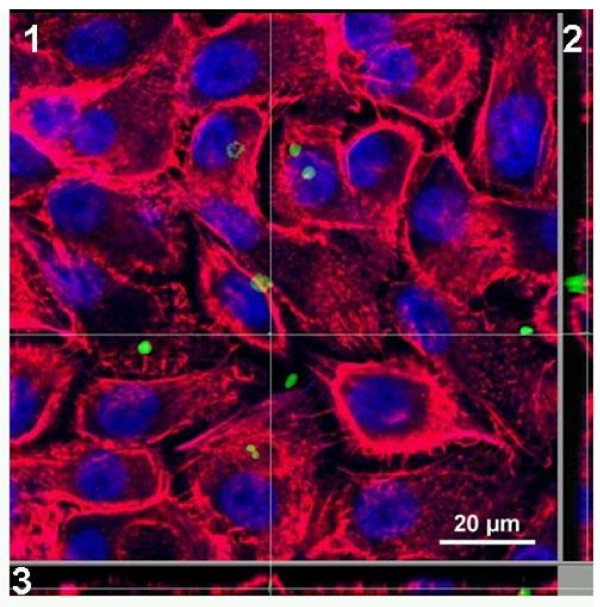
**Confocal microscopy of SPP uptake into normal human bronchial epithelial cells**. Cells were incubated for 8 hours with 5 × 10^6^/cm^2 ^Alexa Fluor 488 labelled subpollen particles (green) in chamber slides. Cell cytoskeletons were stained with an antibody against F-Actin (Rhodamine phalloidin, red) and nuclei staining was performed by TO-PRO-3 Iodide (blue). Three dimensions along the white lines are shown. The intersection displays an internalized SPP.

**Figure 3:**
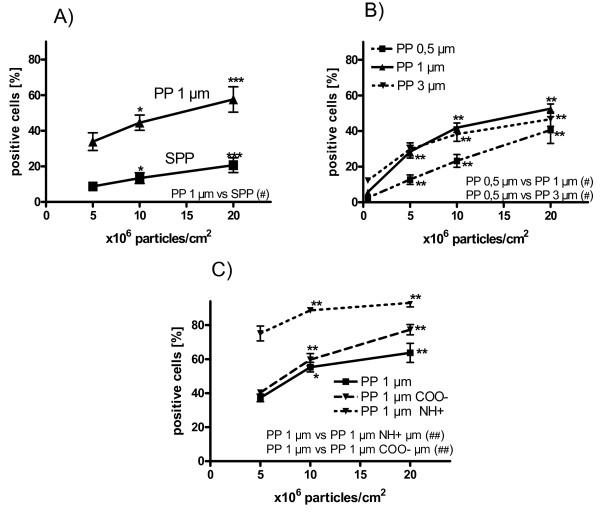
**Association of particles with primary epithelial cells**. A) Percentage of normal human bronchial epithelial cells (NHBE) which participated in uptake and attachment of polydisperse subpollen particles (SPP) and monodisperse 1 μm polystyrene particles (PP) incubated for 8 hours as measured by flow cytometry. B) Percentage of human primary bronchial epithelial cells (HPBEC) which participated in uptake and attachment of PP in different sizes (0.5 μm - 1 μm - 3 μm). C) Percentage of HPBEC which participated in uptake and attachment of PP with different surface charge. Each value represents the mean ± SEM of four to five experiments. * p < 0.05; ** p < 0.01; *** p < 0.001 for within group comparison against 5 × 10^6 ^particles/cm^2 ^; # p < 0.05; ## p < 0.01 for comparison between two groups.

SPP were found attached to and internalized by normal human bronchial epithelial cells (Figure 2). The percentage of commercially available NHBE which took up SPP or had attached SPP (referred to as "positive NHBE"), investigated by flow cytometry, followed a dose-response relationship (Figure 3A). Eight hours of incubation with 5 × 10^6 ^SPP/cm^2 ^led to 8.7 ± 2.3% positive NHBE. Doubling doses of SPP increased the percentage of positive cells to 13.4 ± 3.0% and 20.7 ± 4.2%, respectively (p < 0.05). Incubation of the NHBE with 1 μm polystyrene particles (PP) also showed a significant dose-dependent increase of positive NHBE. Incubation with 5, 10, and 20 × 10^6 ^PP/cm^2 ^led to 33.9 ± 5.0%, 44.5 ± 4.3% and 57.6 ± 7.1% positive NHBE, respectively (Figure 3A). Interestingly, after incubation with the same particle dose, more NHBE were PP-positive than SPP-positive (p < 0.05).

The mean fluorescence intensity (MFI) of SPP-positive or PP-positive NHBE was not changed after incubation with increasing concentrations of SPP or PP, respectively (p > 0.05; data not shown), suggesting that the number of particles per cell remained constant independent from the extra-cellular particle supply.

The effect of particle size on polystyrene particle uptake was studied in fresh human primary bronchial epithelial cells (Figure 3B). PP of different sizes followed a similar dose-response compared to 1 μm PP. Uptake of 0.5 μm PP was significantly lower compared to PP of 1 μm or 3 μm. Overall, the percentage of SPP-positive epithelial cells was lower compared to PP of all sizes tested.

In addition to particle size, we tested the effect of PP surface charge on particle association with HPBEC (Figure 3C). The highest percentage of PP-positive HPBEC was found for the NH-coated PP. The percentage of positive cells increased from 75.1 ± 4.4% after incubation with 5 × 10^6 ^NH-PP/cm^2 ^up to 93.0 ±2.4% after incubation with 20 × 10^6 ^NH-PP/cm^2^. For the COO-PP, respective percentages of 40.1 ± 1.7% and 77.3 ± 3.0% were measured. The percentage of PP positive cells was significantly higher for charged particles compared to plain PP (Figure 3C).

**Figure 4:**
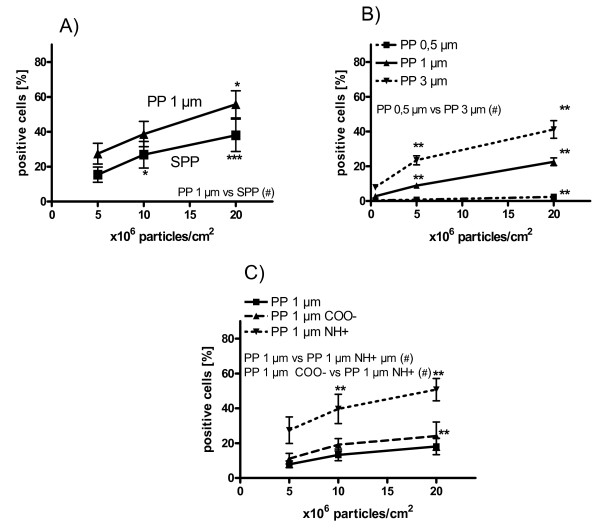
**Association of particles with A549 cells**. A) Percentage of A549 cells which participated in uptake and attachment of polydisperse subpollen particles (SPP) and monodisperse 1 μm polystyrene particles (PP) after 8 hours incubation as measured by flow cytometry. B) Percentage of A549 cells which participated in uptake and attachment of differently sized PP (0.5 μm - 1 μm - 3 μm). C) Percentage of A549 cells which participated in uptake and attachment of PP with different surface charge. Each value represents the mean and SEM from five to eight experiments. * p < 0.05; ** p < 0.01; *** p < 0.001 versus respective 5 × 10^6 ^particles/cm^2 ^value; # p < 0.05 for inter-group comparison.

Alveolar epithelial cells (A549) took up SPP and 1 μm polystyrene particles (Figure 4A). For SPP, a dose dependent increase of particle-positive cells from 15.4 ± 4.4% up to 26.9 ± 7.6% and 38.0 ± 9.3% after incubation with 5, 10 and 20 × 10^6 ^SPP/cm^2 ^(p < 0.05) was detected (Figure 4A). For PP, 27.5 ± 6.0% of the A549 cells participated in particle uptake and adherence after 8 hours incubation with 5 × 10^6 ^PP/cm^2^. The percentage of PP-positive A549 cells was increased up to 38.7 ± 7.2% and 55.7 ± 7.7% (p < 0.05) after duplication and quadruplication of the particle dose. More PP were associated with the cells compared to SPP (p < 0.05). For both, SPP and PP there was a slight but significant increase of MFI with the highest particle dose (p < 0.05; data not shown).

As for HPBEC, the effect of particle size on PP uptake was studied in A549 cells (Figure 4B). PP of different size followed a dose-response. Uptake of 0.5 μm PP was significantly lower compared to 3 μm PP (Figure 4B).

In addition to particle size, we tested the effect of PP surface charge on particle association with A549 cells (Figure 4C). The highest percentage of PP-positive A549 was found for the NH-coated PP (Figure 4C) similar to the effect in HPBEC (Figure 3C). However, the percentage of positive cells increased from 27.4 ± 7.6% after incubation with 5 × 10^6 ^NH-PP/cm^2 ^up to 50.7 ± 6.4% after incubation with 20 × 10^6 ^NH-PP/cm^2 ^which was lower compared to HPBEC.

### Effect of SP-D

**Figure 5:**
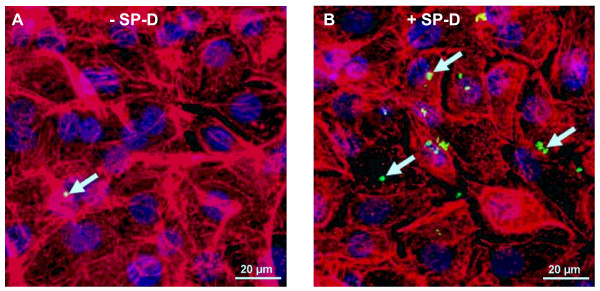
**Representative confocal images visualizing the influence of surfactant protein D (SP-D) on subpollen particle (SPP) uptake and attachment**. Normal human bronchial epithelial cells (NHBE) after incubation with 5 × 10^6 ^SPP/cm^2 ^for 8 hours in A) the absence or B) presence of SP-D (10 μg/ml). White arrows show SPP (Alexa Fluor 488; green); cell cytoskeleton (F-Actin; red); nuclei (TO-PRO-3 iodide; blue).

**Figure 6:**
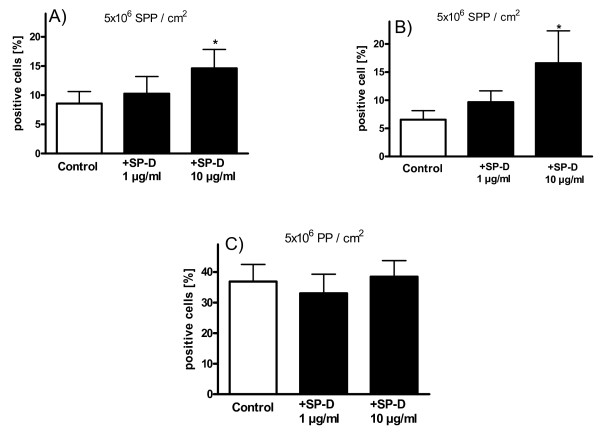
**Effect of surfactant protein D (SP-D) on the percentage of particle-positive human primary bronchial epithelial cells**. Influence of SP-D on the uptake and binding of A+B) subpollen particles (SPP) and C) polystyrene particles (PP) in normal human bronchial epithelial cells (NHBE, A+C) and human primary bronchial epithelial cells (HPBEC, B) that were found particle-positive as measured by flow cytometry. Each value represents the mean ± SEM of five experiments. * p < 0.05 versus control.

After 8 hours incubation, the association of SPP with NHBE was increased by surfactant protein D (Figure 5). Flow cytometric analysis displayed 8.6 ± 2.1% SPP-positive cells after incubation with 5 × 10^6 ^SPP/cm^2^. SP-D (10 μg/ml) increased the percentage of SPP-positive cells significantly to 14.6 ± 3.2% (Figure 6A). A lower concentration of SP-D (1 μg/ml) had no significant effect on the percentage of SPP-positive cells. A similar effect of SP-D on SPP uptake/binding compared to NHBE was seen for HPBEC (Figure 6B). Again, 10 μg/ml SP-D increased the percentage of SPP positive cells while 1 μg/ml SP-D had no significant effect. Interestingly, the number of PP-positive cells was not affected by any of the tested SP-D concentrations (Figure 6C).

In contrast to bronchial epithelial cells, SP-D did not change the percentage of SPP- or PP-positive cells when the alveolar epithelial cell line A549 was used (Table 1).

**Table 1:** Influence of SP-D on percentage of particle-positive A549 cells

Percentage of particle-positive A549-cells			
	**0 μg/ml SP-D**	**1 μg/ml SP-D**	**10 μg/ml SP-D**

**SPP**	15.3 ± 5.0	14.6 ± 5.2	15.8 ± 4.6

**PP 1 μm**	37.3 ± 9.9	41.1 ± 9.5	34.5 ± 8-0

Effect of surfactant protein D (SP-D) on attachment and internalization of 5 × 10^6^/cm^2 ^subpollen particles (SPP) and polystyrene particles (PP) by A549 cells. Each value represents the mean ± SEM from four to eighteen experiments.

The MFI of SPP-positive and PP-positive cells was unchanged when SP-D [1 and 10 μg/ml] was present (data not shown) suggesting that the number of particles per cell remained constant in response to SP-D. In addition, the MFI of positive A549 cells was not changed upon incubation with SP-D (data not shown).

### Differentiation between attachment and internalization of SPP

**Figure 7:**
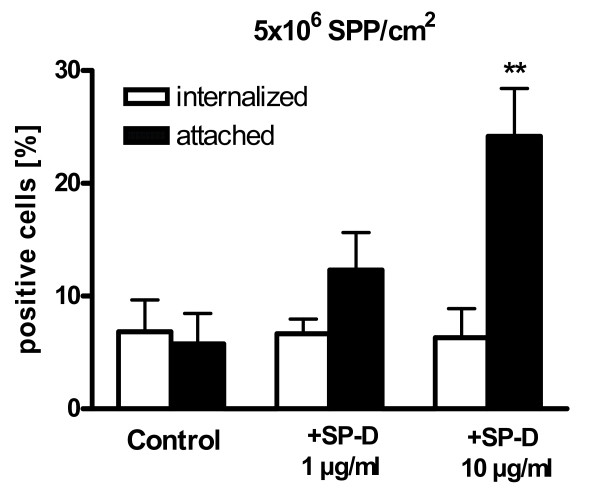
**Differentiation of uptake and attachment of subpollen particles (SPP) in normal human bronchial epithelial cells (NHBE)**. Cells which at least had internalized one SPP were defined as "internalized". These cells could also have attached SPP. Cells which only had attached SPP but no internalized SPP were defined as "attached". At least 10 pictures each with approximately 30 cells were analyzed by confocal microscopy. Each cell was individually assessed for uptake and attachment of SPP. Each value represents the mean ± SEM. ** p < 0.01 versus respective control.

To evaluate whether SP-D increased the uptake of SPP into primary epithelial cells, the attachment of SPP to the cell surface, or both, confocal microscopy was used (Figure 7). After 8 hours incubation with 5 × 10^6 ^SPP/cm^2^, the percentage of NHBE which had internalized at least one SPP was 6.8 ± 2.8%. These cells could also have attached SPP. The percentage of NHBE that had attached at least one SPP, but none internalized was 5.8 ± 3.7%. SP-D [1 and 10 μg/ml] did not change the percentage of epithelial cells which had internalized SPP. However, the percentage of NHBE which had SPP only attached increased to 12.3 ± 3.3% (1 μg/ml SP-D, p = 0.18), and 24.2 ± 4.3% (10 μg/ml SP-D, p < 0.01).

### Inflammatory Response

**Figure 8:**
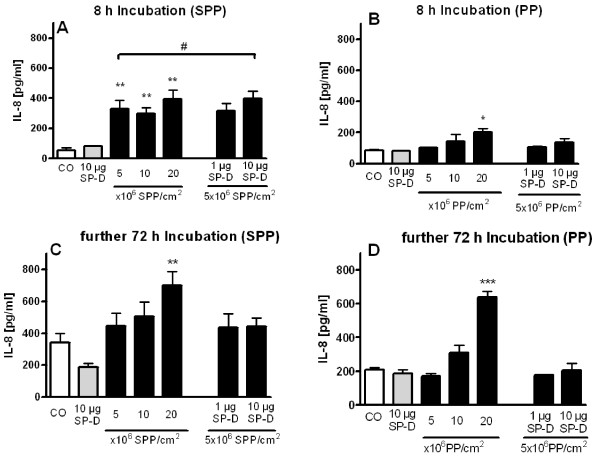
**Interleukin 8 (IL-8) in supernatants of normal human bronchial epithelial cells**. Cells were incubated with particles in the presence of absence of SP-D for 8 hours (A, B) and subsequently cultured in medium for further 72 hours (C, D) using either subpollen particles (SPP, A and C) or polystyrene particles (PP, B and D). Each value represents the mean ± SEM from three experiments. * p < 0.05; ** p < 0.01; *** p < 0.001 versus control (CO). # p < 0.05 versus 5 × 10^6 ^particles/cm^2^. Control represents untreated cells.

Both, SPP as well as PP induced secretion of the chemokine IL-8 into the supernatants of NHBE. After 8 hours incubation with 5, 10, and 20 × 10^6 ^SPP/cm^2^, IL-8 was significantly, but not particle-dose dependently elevated compared to control cells without SPP (329.4 ± 58.0 pg/ml, 298.6 ± 37.9 pg/ml, and 395.0 ± 61.0 pg/ml, respectively versus 55.5 ± 14.7 pg/ml without particles). When SPP (5 × 10^6 ^SPP/cm^2^) were incubated in the presence of SP-D (10 μg/ml), IL-8 secretion was significantly increased which paralleled the additional increase of SPP attachment (Figure 8A).

Incubation with PP induced less IL-8 release compared to SPP. An increase above control levels was only detected for 20 × 10^6 ^PP/cm^2 ^(203.2 ± 22.3 pg/ml). SP-D [1 and 10 μg/ml] did not alter the PP-induced secretion of IL-8 (Figure 8B).

In additional experiments supernatant was removed after 8 hours, epithelial cells were further cultured in fresh medium for 72 hours. The IL-8 secretion was still augmented when cells had been incubated with particles before. Significant increases up to 700.4 ± 86.8 pg/ml after incubation with 20 × 10^6 ^SPP/cm^2 ^(Figure 8C), and up to 638.3 ± 35.8 pg/ml after incubation with 20 × 10^6 ^PP/cm^2 ^(Figure 8D) were detected. Interestingly, the presence of SP-D [1 and 10 μg/ml] during particle exposure (5 × 10^6^/cm^2^) did not modulate IL-8 secretion during the subsequent 72 hours of culture.

Levels of the soluble mediators EOTAXIN, MCP-1, IL-1-α, RANTES and GM-CSF in the supernatant of NHBE were found below or near the limit of quantification and were not modulated after incubation with SPP, PP and SP-D at both time points (data not shown).

## Discussion

The present study demonstrates that primary bronchial epithelial cells and A549 cells take up SPP derived from grass pollen. Previously, it was demonstrated that lung epithelial cells can internalize various particles like polystyrene particles [17] or titanium dioxide particles [18], as well as purified allergens [19]. Furthermore, Malhotra et al. described that A549 cells bind whole pollen grains and that SP-A plays an important role in this interaction [20]. Based on previous observations that SP-D enhanced the uptake of bacteria, fungi, and allergen particles by alveolar macrophages [8,21,22] we hypothesized that SP-D increases attachment and uptake of SPP by epithelial cells.

Importantly, we found that SP-D preferentially promoted attachment of allergen particles to bronchial epithelial cells while the percentage of cells that internalized allergen particles remained unchanged. This might indicate that active allergen uptake by epithelial cells is SP-D independent. This is in contrast to what has been demonstrated for alveolar macrophages where both uptake and binding were increased by SP-D [8]. While uptake of SPP by macrophages was augmented at SP-D concentrations between 0.25-5 μg/ml [8], a significant increase of SPP attachment to epithelial cells was only present at a higher SP-D dose of 10 μg/ml. We assume, that SP-D concentrations of 1 μg/ml and 10 μg/ml as used in our study reflect a physiologically relevant range. In the rat lung, the estimated concentration of SP-D in the hypophase ranges from 36 μg/ml to 216 μg/ml [23]. In humans, the concentration of SP-D in lavage fluid was investigated to be around 1.3 μg/ml [24] and can be found increased in various diseases [24,25].

It is important to note that microparticles are usually not taken up by epithelial cells *in vivo *but are predominantly phagocytosed by macrophages [26]. The increased attachment of SPP to the surface of epithelial cells by SP-D could lead to a better presentation of SPP to resident macrophages which might facilitate SPP uptake by macrophages. This could be a further mechanism how SP-D facilitates particle uptake by macrophages besides the direct effect of SP-D to augment particle uptake by macrophages [8].

Potential mechanisms how SP-D augments particle uptake and binding include opsonisation and activation of cellular receptors. SP-D binds to different carbohydrates (e.g. glucose, fucose or mannose [27]) that can be found on the surface of SPP [28]. It is likely that the interaction of SP-D with these carbohydrates facilitates opsonisation by SP-D and thereby improves the attachment of SPP to epithelial cells. Since blank PP exhibit no surface coating, binding of SP-D and thereby aggregation of PP was not observed (data not shown).

Several receptors on epithelial cells are candidates for an interaction with SP-D. Important receptors are e.g. the calreticulin-CD91 complex or Sirp-alpha [7,29]. However, our study did not attempt to study the cellular mechanisms of the SP-D effect but rather to investigate and compare different particles and epithelial cells.

At similar particle concentrations, the percentage of PP-positive cells (1 μm) was significantly higher than the percentage of SPP-positive cells. A reason for this could be the different size of the particles. PP (1 μm) used in this study were monodisperse with an exact diameter of 1 μm while the allergen particles had a polydisperse size distribution. It is known that both uptake of particles into cells [30-32] and translocation through the epithelium are size dependent phenomena [33,34]. In addition, particle size determines the uptake mechanisms. Accordingly, for the relatively large particles that we used in the present study mainly phagocytosis or macropinocytosis play a role [35]. Our results demonstrate that 1 μm PP are associated with epithelial cells to a higher degree than smaller PP (0.5 μm). Importantly, a large number of the SPP were lower than 1 μm (Figure 1). Therefore, we assume that this size dependency is one reason for the lower association of SPP with epithelial cells compared to PP.

A second reason could be related to differences in surface charges of the particles. Our results indicate that charged particles are to a higher degree associated with epithelial cells (Figure 3 and 4). The highest particle-cell-association was found for positively charged particles but negatively charged particles were also more associated with epithelial cells compared to plain particles. SPP were found to have a very negative potential (Table 2). Therefore, it is unlikely that differences of surface charges between SPP and PP can explain the difference in particle-cell-association.

**Table 2:** Zeta potential of various particles

	Zeta Potential (mV)
**SPP**	- 50.2 ± 1.9

**PP 0,5 μm (Polysciences)**	-21,0 ± 2.6

**PP 1 μm (Polysciences)**	-32,6 ± 0.6

**PP 3 μm (Polysciences)**	-38,6 ± 0.8

**PP 1 μm**	-31,6 ± 0.5

**PP 1 μm -COOH**	-38,7 ± 1.6

**PP 1 μm -NH**	6,8 ± 0.3

Zeta potentials (mV) of the used particles are shown. Each value represents mean ± SEM from three experiments. PP (Polysciences) were provided from Polysciences. The other PP were provided from Invitrogen.

Finally, SPP and PP differed in their surface composition. While PP were uncoated or had NH^+ ^or COO^- ^groups, the surface of the SPP usually exhibit complex glycolipids at their outer surface [36]. Unfortunately, we were unable to modify the surface of PP to be composed of more complex surface molecules similar to SPP in order to directly determine the effect of surface composition. It is very likely that the surface composition of particles determine the uptake because differences in particle uptake due to surface composition or surface coating were previously shown for other particles [37,38]. Therefore, we conclude that both particle size and surface composition attribute to the observed difference in particle uptake between SPP and PP by epithelial cells.

We have used primary human bronchial epithelial cells that are commercially available (NHBE) as well as primary human bronchial epithelial cells that were harvested by bronchoscopy from healthy volunteers (HPBEC). In order to compare these two cells, we investigated the effect of SP-D on binding/uptake of SPP (Figure 6A &6B). Importantly, HPBEC exhibited a similar SPP association and SP-D effect compared to commercially available cells. In contrast, A549 cells revealed a different behaviour. In the absence of SP-D SPP binding and uptake by A549 cells was higher compared to the primary cells, but SP-D showed no effect of modulating the uptake and attachment of SPP. We can exclude that endogenous production of SP-D by A549 cells accounts for this difference because we did not detect SP-D in the cell culture supernatant by ELISA (data not shown). More likely, the observed differences between the cells may be due to their origin. A549 cells are alveolar type II pneumocyte-like cells that were derived from a human pulmonary adenocarcinoma [39] while the primary cells used in this study were not immortalized and of bronchial origin. In addition, A549 cells are proliferating cells whereas the lifespan of the primary cells is limited. Importantly, previous work has shown that alveolar and bronchial cells can differ in several characteristics like capabilities of particle uptake [40,41] or receptor expression on the cell surface, which are involved in uptake of various substances [42].

Admittedly, inhaled particles act directly on the epithelial cells at the air-liquid interface and not primarily through the liquid cell culture medium as in our *in vitro *experiments. However, after deposition at the air-liquid interface the particles are displaced to the epithelial lining layer by pulmonary surfactant and thereby interact with dissolved proteins like the surfactant protein D. Therefore, our assay system is at least capable to demonstrate the interaction of particles with dissolved SP-D and the resulting consequences for association with epithelial cells.

The release of IL-8 by epithelial cells is commonly seen upon contact with allergens [43-46] or particulate material [47,48]. Accordingly, we have observed an increased IL-8 release after exposure to allergen particles and even after exposure to PP (although only at the highest concentration which might be an unspecific particle overload phenomenon). IL-8 differs from other cytokines in its ability to specifically attract and activate neutrophil granulocytes, which is a pro-inflammatory action. However, in allergic disease IL-8 may also play an anti-inflammatory role since it inhibits histamine release from basophils [49] and antagonizes IgE production by B cells [50]. The increased attachment of allergen particles to epithelial cells in the presence of SP-D was accompanied by a further increase of IL-8 release. Thus, SP-D may positively modulate the allergic inflammation by the enhancement of IL-8 release from epithelial cells in the presence of allergen. This mechanism, although not yet proven in allergic subjects or animal models, may add to further anti-inflammatory effects of SP-D that were observed during the allergic inflammation [51].

In summary, our data demonstrate that primary bronchial epithelial cells bind and internalize allergen particles derived from grass pollen which is paralleled by an increased secretion of IL-8. SP-D increased the attachment of allergen particles to bronchial epithelial cells and further increased the secretion of IL-8 which may add to the anti-inflammatory effects of SP-D in allergic diseases.

## Conclusions

Human primary bronchial epithelial cells internalize and attach allergen particles which lead to increased IL-8 secretion. SP-D promotes attachment of SPP to epithelial cells and may thus be involved in the clearance of deposited allergen particles as well as in the inflammatory response to inhaled allergen.

## List of abbrevations

CRD: Carbohydrate recognition domain; HPBEC: Human primary bronchial epithelial cells; IL: Interleukin; MFI: Mean fluorescence intensity; NHBE: Normal human bronchial epithelial cells; PBS: Phosphate buffered saline; PP: Polystyrene particle; SP: Surfactant Protein; SPP: Subpollen particles;

## Competing interests

The authors declare that they have no competing interests.

## Authors' contributions

CS planned the concept and study design, performed the experiments, interpreted the results and wrote major parts of the manuscript. VJE planned the concept and study design, performed the experiments, interpreted the results and wrote major parts of the manuscript. CW performed the experiments with the different PP sizes and surface charges. HDL, MN, AB, and NK made substantial contributions to the analysis and interpretation of the data. JMH planned the concept and study design, made substantial contributions to the analysis and interpretation of the data and wrote major parts of the manuscript. All of the authors have critically read the manuscript and approved its submission.
